# Inflammatory Microenvironment Accelerates Bone Marrow Mesenchymal Stem Cell Aging

**DOI:** 10.3389/fbioe.2022.870324

**Published:** 2022-05-12

**Authors:** Xin Peng, Xin Zhou, Ying Yin, Beibei Luo, Yang Liu, Cheng Yang

**Affiliations:** ^1^ Department of Stomatology, Union Hospital, Tongji Medical College, Huazhong University of Science and Technology, Wuhan, China; ^2^ School of Stomatology, Tongji Medical College, Huazhong University of Science and Technology, Wuhan, China; ^3^ Hubei Province Key Laboratory of Oral and Maxillofacial Development and Regeneration, Wuhan, China

**Keywords:** aging-related skeletal diseases, stem cell therapy, inflammatory microenvironment, bone marrow mesenchymal stem cell, aging

## Abstract

MSC senescence is considered a contributing factor in aging-related diseases. We investigated the influence of the inflammatory microenvironment on bone marrow mesenchymal stem cells (BMSCs) under aging conditions and the underlying mechanism to provide new ideas for stem cell therapy for age-related osteoporosis. The BMSCs were cultured until passage 3 (P3) (young group) and passage 10 (P10) (aging group) *in vitro*. The supernatant was collected as the conditioned medium (CM). The young BMSCs were cultured in the CM of P3 or P10 cells. The effects of CM from different groups on the aging and stemness of the young BMSCs were examined. A Quantibody^®^ mouse inflammation array on serum extracts from young (aged 8 weeks) and old (aged 78 weeks) mice was performed, and differentially expressed factors were screened out. We discovered that the CM from senescent MSCs changed the physiology of young BMSCs. Systemic inflammatory microenvironments changed with age in the mice. In particular, the pro-inflammatory cytokine IL-6 increased, and the anti-inflammatory cytokine IL-10 decreased. The underlying mechanism was investigated by GO and KEGG analyses, and there was a change in the JAK-STAT signaling pathway, which is closely related to IL-6 and IL-10. Collectively, our results demonstrated that the age-related inflammatory microenvironment has a significant effect on the biological functions of BMSCs. Targeted reversal of this inflammatory environment may provide a new strategy for stem cell therapy to treat aging-related skeletal diseases.

## Introduction

Osteoporosis is a common aging-related disease. Currently, the clinical treatment of osteoporosis is mainly medical therapy, including bisphosphonates, calcitonin, and SERM (Khosla and Hofbauer, 2017). However, these drugs often bring about side effects, such as atypical femoral fractures and osteonecrosis of the jaw (Khosla and Hofbauer, 2017), and their long-term efficacy is not fully confirmed. Studies have shown that cell therapy based on bone marrow mesenchymal stem cells is suitable for the treatment of osteoporosis (Aghebati-Maleki et al., 2019).

As osteoporosis is related to a decline in the number and function of osteoblasts, substantial evidence indicates that the transplantation of stem cells with osteogenic differentiation ability has the ability to reverse bone demineralization (Paspaliaris and Kolios, 2019). Microenvironment regulation is one of the main mechanisms of stem cell–mediated anti-aging and the treatment of aging diseases (Paspaliaris and Kolios, 2019). Stem cells can secrete various biologically active proteins, including growth factors and cytokines, and act on different cells in tissues and organs through an intercellular signal transduction system (Galkowski et al., 2017).

Increasing evidence indicates that chronic inflammation in elderly individuals (inflammaging) is intensively associated with many aging-related diseases, such as Alzheimer’s disease, atherosclerosis, heart disease, type II diabetes, and cancer. Inflammatory factors affect bone remodeling under physiological conditions, such as fracture repair. Inflammation is closely associated with osteoclast differentiation and bone loss; however, it has become clear that there is an important relationship between inflammation and bone formation (Adamopoulos, 2018). However, whether we can adjust this inflammatory microenvironment to improve the function of bone marrow mesenchymal stem cells to provide new strategies for stem cell therapy to treat aging-related skeletal diseases remains to be shown.

This study aimed to evaluate the effects of the inflammatory microenvironment on BMSC proliferation, differentiation, and stemness. We also used the inflammation array to examine the serum of mice in different age groups to explore changes in the related signaling pathways. After being treated with P10 MSC CM, BMSCs’ stemness was reduced compared with that of the BMSCs treated with P3 MSC CM. A Quantibody^®^ mouse inflammation array was used to examine the serum of mice in different age groups to explore changes in the related signaling pathways. With increasing age, an inflammatory microenvironment occurred, and IL-6 and IL-10 had the most significant changes; IL-6 increased and IL-10 decreased. The immunohistochemical analysis showed that the local environment in the bone marrow had the same change. Furthermore, ELISA was used to verify that IL-6 was increased and IL-10 was decreased in P10 MSC CM. The mechanism was evaluated through the GO and KEGG analyses, and the JAK-STAT signaling pathway was changed. Therefore, our findings suggested that the increase in IL-6 and the decrease in IL-10 in the microenvironment reduced BMSCs’ stemness through the JAK-STAT signaling pathway, leading to decreased osteogenic differentiation. Additionally, these results suggested that inflammaging was a major contributor to the decline in the regenerative capacity of the skeleton, suggesting a potential therapeutic strategy for preventing bone loss in osteoporosis. Perhaps targeting specific inflammatory mediators to control age-related inflammation could have beneficial therapeutic effects in treating age-related diseases (Lasry and Ben-Neriah, 2015).

## Materials and Methods

### Ethics

All animal care and experimental protocols were approved by the Institutional Animal Care and Use Committee of Tongji Medical College, Huazhong University of Science and Technology (Wuhan, China, S780), which were according to the National Institutes of Health Guidelines for the Care and Use of Laboratory Animals.

### Animals

All male C57BL/6J mice (aged 8 weeks or 78 weeks) were purchased from Beijing Vital River Laboratory Animal Technology Co., Ltd. All these mice were raised in the specific pathogen-free (SPF) animal center of Tongji Medical College, Huazhong University of Science and Technology (Wuhan, China). The mice were maintained at a room temperature of 23 ± 1°C and 55 ± 5% humidity under a 12 h light/dark cycle and supplied with sterilized food and water. The irradiated corncob bedding was changed every week.

### Isolation and Cultivation of Rat BMSCs

Sprague–Dawley rats (aged 3 weeks, male, weighing approximately 60–80 g) were used for collecting and culturing the BMSCs for the experiments *in vitro*.

The bone marrow of femurs and tibias was drawn out by flushing with a complete medium. The bone marrow cells of each rat were seeded in two 25-cm^2^ tissue culture flasks containing DMEM/F-12 medium (HyClone, Logan, UT, United States), 10% fetal bovine serum (FBS) (Gibco, United States), and 1% penicillin-streptomycin solution (Beyotime, Shanghai, China). The BMSCs were maintained at 37°C in a 5% CO_2_ humidified incubator. By changing the medium every 2 days, non-adherent cells were removed and adherent cells were cultured until confluent to 80%.

### Cell Proliferation and Apoptosis Assay

#### CCK8 Assay

At a density of 1 × 103 cells/well, the BMSCs were seeded in 96-well plates. Cell counting kit-8 (Dojindo, Kumamoto, Japan) was used to measure cell proliferation. As described previously, 10 µl of CCK-8 solution per 100 µl medium was added to every well, and the plates were incubated for 1 h after being mixed. The OD value was recorded by using a microplate reader (EnSpire, PerkinElmer, United States) at 450 nm. We performed this assay on day 1, day 3, day 5, and day 7.

### EdU Assay

The cells were cultured on the slides (round, thickness: 0.2 mm, and diameter: 15 mm). Cell proliferation was analyzed using the BeyoClick™ EdU Cell Proliferation Kit (Beyotime, Shanghai, China) with Alexa Fluor 488, according to the protocol. Every well was treated with 500 µl of medium containing 10 µM EdU. After incubation at 37°C, with 5% CO_2_ for 2 h, the cells were fixed with 4% paraformaldehyde at room temperature for 15 min and incubated with 0.3% Triton X-100 in PBS for 10–15 min. The cell nuclei were stained with Hoechst 33342. Images of three randomly selected areas of each group were taken using a laser scanning confocal microscope (A1si+/A1Rsi+, Nikon, Japan). The percentage of proliferative cells was calculated by the number of EdU-positive cells compared with all cells in different fields as already shot.

### TUNEL Assay

Cell apoptosis was evaluated by using the One-Step TUNEL Apoptosis Assay Kit (Beyotime, Shanghai, China). The apoptotic cells were detected using a fluorescence microscope (Olympus, Japan). Images of three randomly selected areas of each group were taken.

### Senescence Assay

The senescence β-galactosidase staining kit was used to test the activity of senescence-associated β-galactosidase (SA-β-Gal) (Beyotime, Shanghai, China). According to the protocol, the senescent cells will be stained.

### Osteogenic and Adipogenic Differentiation Assay

To induce osteogenic differentiation, the BMSCs were cultured in a commercially available osteogenic medium (Cyagen, Soochow, China) for 14 days. Thereafter, the cells were washed twice with phosphate-buffered saline (PBS), fixed in 4% paraformaldehyde for 15 min at room temperature, and stained with alizarin red (Servicebio, Wuhan, China) for 30 min. After rinsing the cells 2–3 times with PBS, the calcified nodules were visualized under an inverted microscope (Olympus, Tokyo, Japan). Similarly, the BMSCs were cultured in a commercially available adipogenic medium (Cyagen, Soochow, China) for 2 weeks. Subsequently, the cells were washed twice with PBS, fixed in 4% paraformaldehyde for 15 min at room temperature, then washed with PBS, and stained with 2% oil red O (Servicebio, Wuhan, China) for 1 h at room temperature. The BMSCs were washed again with PBS. The intracellular lipid droplets were visualized under a microscope (Olympus, Tokyo, Japan).

### RNA Extraction and Real-Time RT-PCR of mRNA

Total RNA was extracted from cells using the RNA isolater Total RNA Extraction Reagent (Vazyme, Jiangsu, China), following the manufacturer’s protocol. Complementary DNA was generated using the HiScript^®^ II Q RT SuperMix for qPCR (+ gDNA wiper) (Vazyme, Jiangsu, China). The qRT-PCR was performed in a 96-well plate with ChamQ SYBR qPCR Master Mix (Vazyme, Jiangsu, China), and it was performed on an ABI StepOnePlus real-time PCR instrument (Applied Biosystems). The mRNA levels of the target genes were normalized against that of GAPDH, which served as an internal control.

### Western Blot Analysis

The cells were lysed by RIPA buffer supplemented with a protease inhibitor cocktail. An equal amount of proteins (containing 30 mg protein lysate) were loaded into 12% polyacrylamide gel for SDS-PAGE electrophoresis and transferred to 0.45 µm or 0.22 µm polyvinylidene fluoride membranes, which were subsequently incubated with 5% skim milk for 1 h and then probed with rabbit antibodies against Perilipin 1 (1:2000, ab3526, Abcam), FABP4 (1:500, 15872-1-AP, Proteintech), SOX2 (1:500, A0561, ABclonal), and GAPDH (1:10000, 10494-1-AP, Proteintech). The enhanced chemiluminescence substrate reagent (Millipore, Billerica, MA, United States) was used to detect immunoreactive protein bands. The gray values of the blots were analyzed by ImageJ software. The value of each blot was normalized to the value of GAPDH.

### Quantibody^®^ Mouse Inflammation Array 1

The RayBiotech Quantibody^®^ Array, multiplexed sandwich ELISA-based quantitative array platform, enables us to accurately measure the concentration of multiple cytokines simultaneously. In detail, the serum samples and standards were incubated on the array overnight at 4°C. The slides were washed with a proprietary buffer and then incubated with a detection antibody at room temperature for 2 h. The slides were then washed and incubated with the streptavidin-conjugated Cy3 equivalent dye at room temperature for 1 h. Thereafter, the slides were washed a final time and then dried thoroughly before scanning with a laser scanner using the Cy3 excitation profile. Then, we calculated and analyzed the cytokine concentration.

### Enzyme-Linked Immunosorbent Assay

Supernatants were collected from the BMSCs. The concentrations of IL-6 and IL-10 in the supernatant were determined by using commercial ELISA kits (NEOBIOSCIENCE, China), according to the manufacturer’s instructions. The absorbance was measured by using a microplate reader at 450 nm.

### Histological Procedure and Immunohistochemical Staining

The femur specimens were fixed in 4% paraformaldehyde for 24 h, decalcified with 10% EDTA in PBS for 3 weeks, embedded in paraffin, and sectioned to a 5-µm thick specimen. Sections were stained by hematoxylin and eosin (H&E) staining. For immunohistochemical staining, the sections were placed in sodium citrate buffer for 20 min at 90ºC for antigen retrieval and incubated with monoclonal anti-mouse IL-6 and IL-10 antibodies overnight at 4°C.

### Statistical Analysis

All the data were displayed as mean ± standard deviation (SD). ImageJ software was used for quantitative protein analysis. Data were evaluated by GraphPad Prism 7.00 software using Student’s *t*-test or one-way ANOVA followed by Tukey’s *post hoc* test. A *p*-value < 0.05 was considered significant.

## Results

### With Increasing Age, the Probability of Age-Related Osteoporosis Increases.

**FIGURE 1:**
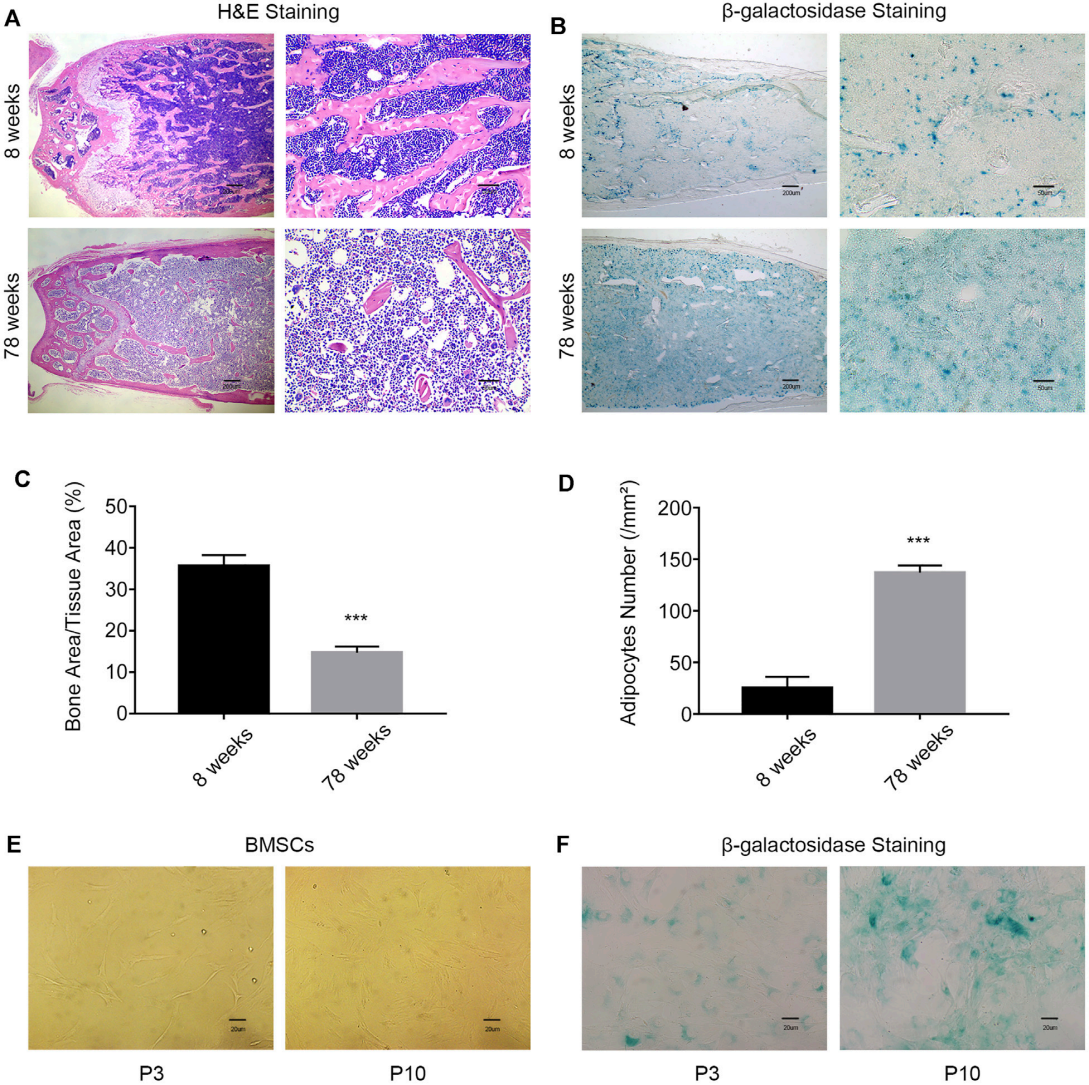
With increasing age, the probability of age-related osteoporosis increases. **(A)** Representative images of H&E staining of the femurs of 8-week-old and 78-week-old WT mice. Scale bars, 200 and 50 µm. **(B)** β-Galactosidase staining of femur sections from 8-week-old and 78-week-old WT mice. Scale bars, 200 and 50 µm. **(C)** Percentage of the bone mass in femur sections from 8-week old and 78-week-old WT mice (*n* = 3 and ***p < 0.001). **(D)** Enumeration of bone adipocytes in the femurs of 8-week-old and 78-week-old WT mice (*n* = 3 and ***p < 0.001). **(E)** Morphology of MSCs was observed under an inverted microscope. Scale bar, 20 µm. **(F)** β-gal staining (blue: senescent cells) of MSCs was observed under an inverted microscope. Scale bar, 20 μm. P3, early passage; P10, late passage.

Clinically, older patients have a higher incidence of fractures than younger patients. Hematoxylin–eosin (HE) staining of femur slices was used to assess the bone mass and fat content in young (8-week-old) mice and aged (78-week-old) mice. The bone mass in the aged group was significantly lower than that in the young group. Conversely, the fat content in the aged group was higher than that in the young group (Figures 1A,C,D). The activity of β-galactosidase was enhanced in the bone marrow cavity in the aged group, which indicated an increased percentage of the senescent cells in aged mice (Figure 1B). After long-term passage *in vitro*, the BMSCs in the aging group (P10) showed changes in morphology, including larger and flatter cell features (Figure 1E), and enhanced β-galactosidase activity (Figure 1F). These results indicated that the number of senescent cells increases with aging.

### CM From Senescent MSCs Changes the Physiology of Young BMSCs

**FIGURE 2:**
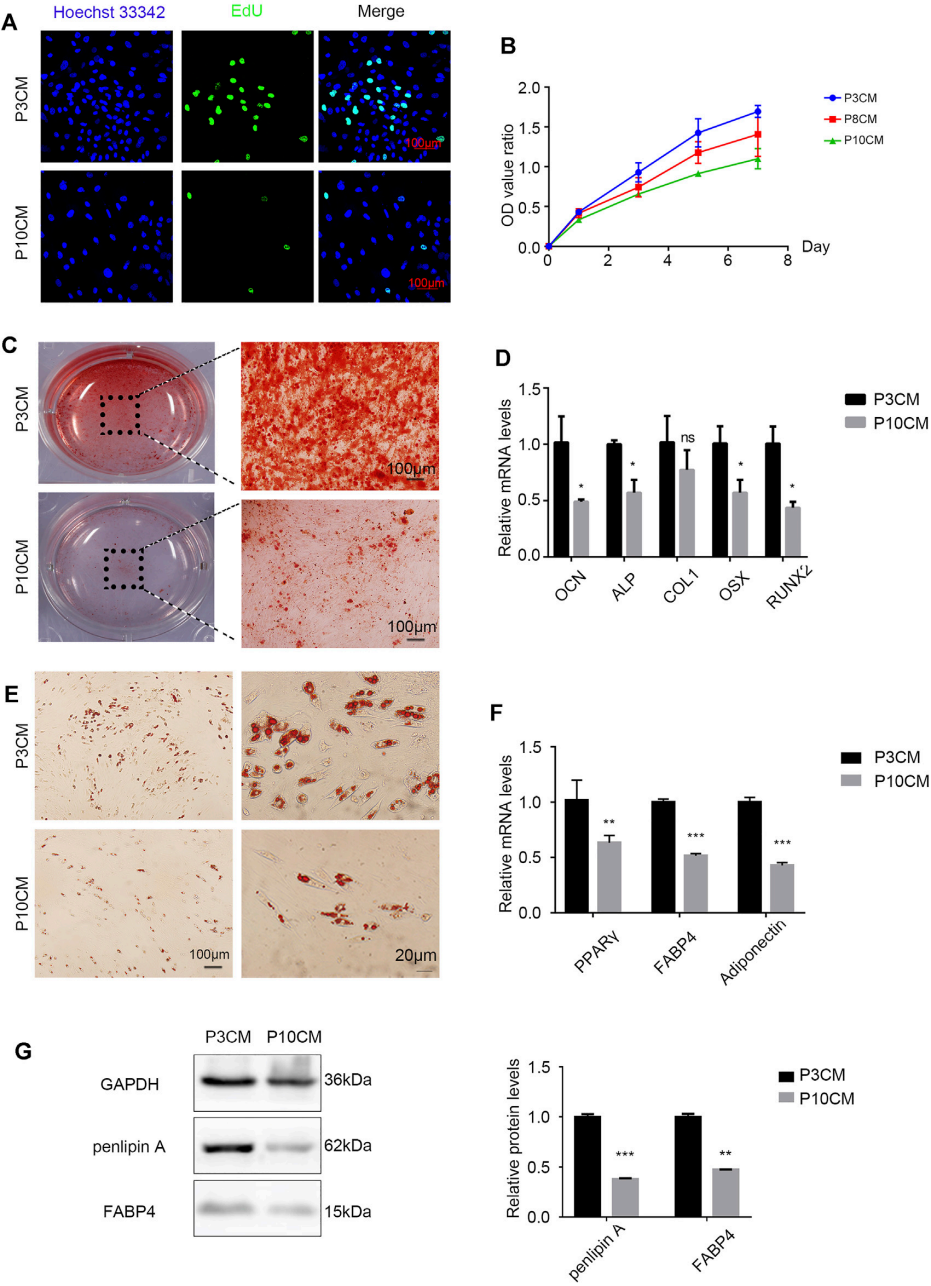
CM from the senescent MSCs changes the physiology of young BMSCs. **(A)** EdU staining of the BMSCs cultured with P3 CM or P10 CM (scale bar: 100 μm). **(B)** Growth curves of the BMSCs cultured with P3 CM or P10 CM. **(C)** Alizarin red staining shows the osteogenic induction of the young BMSCs cultured with P3 CM or P10 CM (*n* = 3, magnification: × 100, and scale: 100 µm). **(D)** Real-time PCR showing the expressions of OCN, ALP, COL1, OSX, and RUNX2 (*n* = 3, *p < 0.05, ns., and *p* > 0.05). **(E)** Oil red O staining of the young BMSCs cultured with P3 CM or P10 CM. **(F)** Real-time PCR showing the expressions of PPARγ, FABP4, and adiponectin (*n* = 3, **p < 0.01, and ***p < 0.001). **(G)** Western blot analysis of lipid-related indicators such as FABP4 and perilipin A (*n* = 3, **p < 0.01, and ***p < 0.001).

Stem cell senescence is caused by a combination of intrinsic and irreversible changes through circulating effectors or factors secreted by local stem cell niches (Severino et al., 2013). To determine whether extracellular factors produced by the senescent cells can alter the physiological characteristics of stem cells, proliferation and the differentiation capacity of young MSCs cultured with conditioned medium (CM) from the P3 and P10 BMSCs were evaluated. When P3 and P10 BMSC cultures reached an 80% confluency, the serum-free base medium was added after discarding the original medium, and the supernatant (CM) was collected and stored at −80°C for 48 h. As expected, the positive rate of EdU staining in the CM-P10 cultured cells was lower than that in the CM-P3 cultured cells (*n* = 3 and *p* < 0.01) (Figure 2A). The proliferation rate of BMSCs was gradually decreased when the cells were cultured in CM-P10 compared with those cultured in CM-P3 (Figure 2B). Alizarin red staining revealed that there was an evident decrease in osteoblast differentiation in the mBMSCs cultured in CM-P10 compared with those cultured in CM-P3 (Figure 2C, Supplementary Figure S1). Furthermore, the RT–PCR analysis indicated that the expressions of osteogenic markers, such as OCN, ALP, Runx2, and OSX, were decreased when the cells were cultured in CM-P10 (*n* = 3 and *p* < 0.05) (Figure 2D). Oil red O staining indicated that the number of lipid droplets in the CM-P10 group was significantly reduced compared to that in the CM-P3 group (Figure 2E, Supplementary Figure S1). Moreover, the expressions of PPARγ, Fabp4, adiponectin, and perilipin A, which are adipogenic markers, were decreased in the CM-P10 group (Figures 2F,G). The adipogenic capacity was decreased in the CM-P10 group. Taken together, these data indicate that the proliferation and differentiation capacity of BMSCs is significantly decreased by treatment with a conditioned medium of senescent cells.

With aging, the external microenvironment has a greater impact on the proliferation capacity of the BMSCs. However, the TUNEL assay showed that the external environment in aging had no obvious effect on BMSC apoptosis (Supplementary Figure S2). This result shows that the external environment in aging mainly reduces the number of BMSCs by reducing the proliferation of the BMSCs rather than inducing apoptosis.

### CM From Senescent MSCs Decreases Stemness and Triggers Senescence in Young BMSCs

**FIGURE 3:**
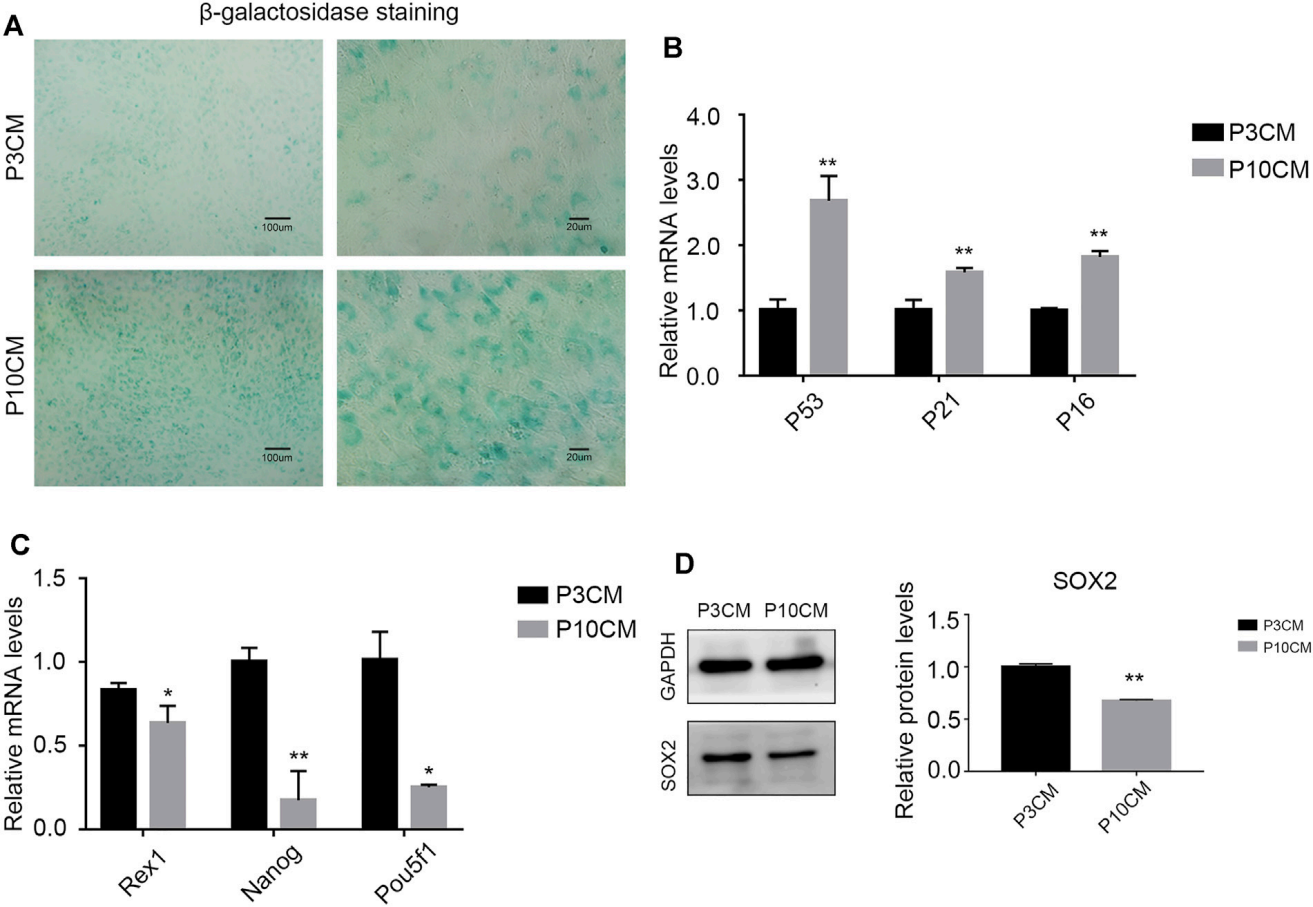
CM from the senescent MSCs decreases stemness and triggers senescence in young BMSCs. **(A)** β-galactosidase staining of the BMSCs cultured with P3 CM or P10 CM. (Magnification: × 100, scale bar: 100 μm; magnification: × 400, scale bar: 20 µm). **(B)** Real-time PCR results showing the expressions of P53, P21, and P16 and other aging-related indicators (*n* = 3, **, and *p* < 0.01). **(C)** Real-time PCR results showing the expression of the stemness-related indicators Rex1, Nanog, and Pou5f1 (*n* = 3, *p < 0.05, and **, *p* < 0.01). **(D)** Western blot analysis verified the protein expression level of SOX2, a stemness-related indicator (*n* = 3 and **p < 0.01).

We cultured the BMSCs with CM-P3 and CM-P10 *in vitro*. The activity of β-galactosidase in the CM-P10 group was enhanced (Figure 3A). RT–PCR showed that the transcription level of the aging-related indicators P53, P21, and P16 was increased (Figure 3B). We know that the external microenvironment in aging can accelerate aging in young BMSCs. In addition, we measured the expression levels of Rex1, Nanog, and Pou5f1, which are related to cell stemness, and found that the external microenvironment in aging could reduce the stemness of BMSCs (Figure 3C). Western blot analysis verified that the protein expression level of SOX2, a dryness-related indicator, decreased after cells were cultured with CM-P10 (Figure 3D). These results demonstrated that the external environment in aging decreased the proliferation and stemness of the BMSCs and promoted BMSC senescence.

### Increased Inflammation in the Microenvironment With Age

**FIGURE 4:**
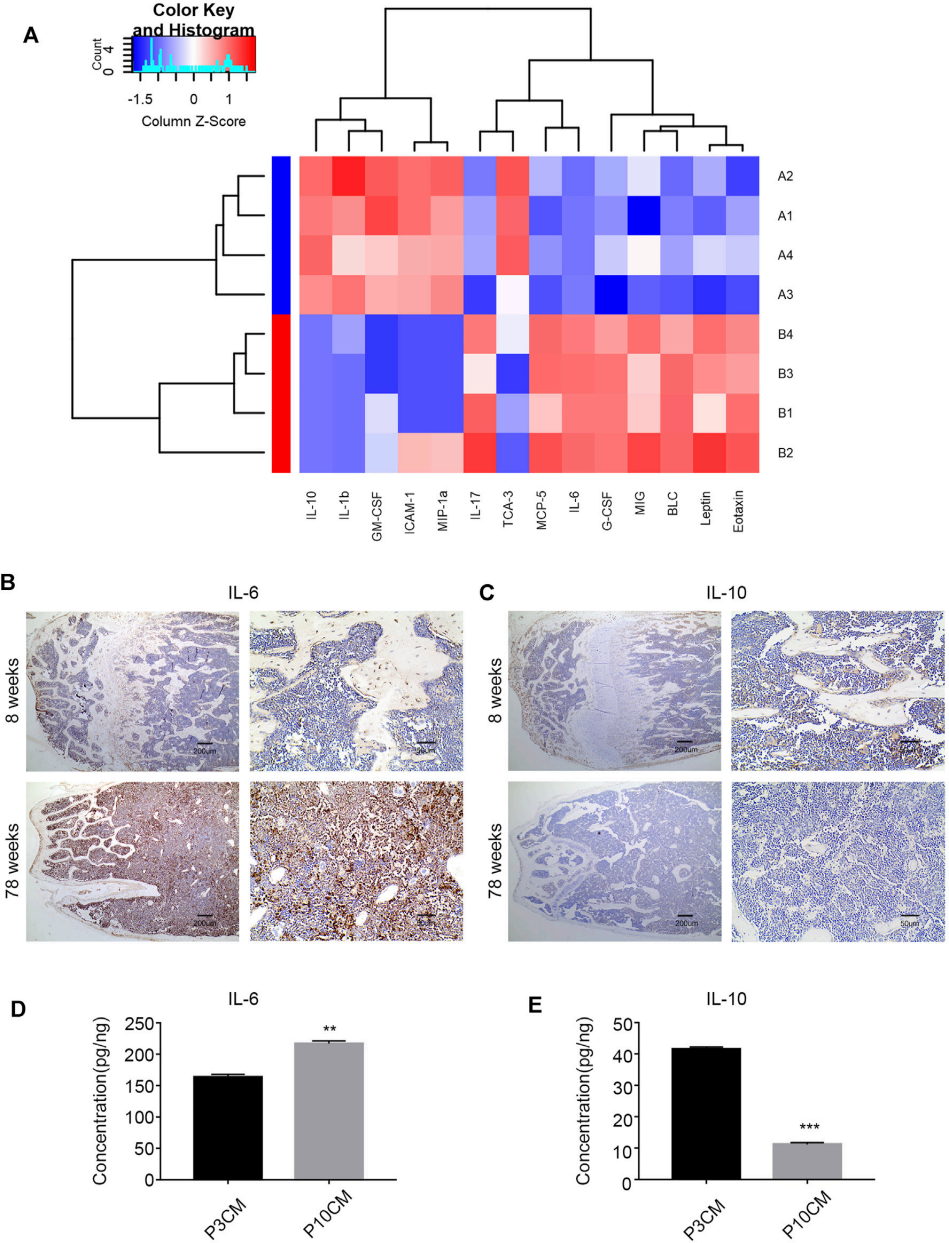
Increased inflammation in the microenvironment with age. **(A)** Heatmap analysis of inflammatory factors in the serum of young mice (8 weeks old) and old mice (78 weeks old). (Group A: young group, Group B: aged group, *n* = 4). **(B)** Immunohistochemical analysis of the IL-6 expression in the bone marrow cavities of mice in different age groups. **(C)** Immunohistochemical analysis of the IL-10 expression in the bone marrow cavities of mice in different age groups. **(D)** ELISA was used to verify the level of IL-6 in the cell supernatant of the young group (P3 CM) and senescent group (P10 CM) (*n* =3, **p < 0.01). **(E)** ELISA was used to verify the level of IL-10 in the cell supernatant of the young group (P3 CM) and senescent group (P10 CM) (*n* = 4 and ***p < 0.001).

Chronic inflammation is associated with many diseases, and inflammatory factors play a key role. Studies have shown that elderly individuals are in a chronic, low-level subclinical pro-inflammatory state (Chung et al., 2011; Cevenini et al., 2013; Lin et al., 2017). To explore the relationship between the age-related inflammatory microenvironment and osteoporosis, we performed a Quantibody^®^ mouse inflammation array on serum extracts from young (aged 8 weeks) and old (aged 78 weeks) mice. The expressions of pro-inflammatory cytokines and chemokines were increased in the serum of old mice, and 14 cytokines reached statistical significance (Figure 4A). The cytokines with significant differences are shown in Supplementary Table S1. Among them, the pro-inflammatory factor IL-6 was increased, and the anti-inflammatory factor IL-10 was decreased most obviously, suggesting a trend of increased inflammation.

To evaluate the changes in IL-6 and IL-10 in the bone marrow microenvironment during aging, we studied femur sections from young (8-week-old) and aged (78-week-old) male C57BL/6 mice. We analyzed the levels of IL-6 and IL-10 in the bone marrow in different age groups by immunohistochemistry. The results showed an increase in IL-6 and a decrease in IL-10 in the aged group (Figures 4B,C). We confirmed this trend in CM by ELISA, and the results revealed increases in the pro-inflammatory cytokine IL-6 and decreases in the anti-inflammatory cytokine IL-10 in CM-P10 (,Figures 4D,E). In summary, we demonstrated that the age-associated shift in the inflammatory microenvironment is associated with an increase in the pro-inflammatory cytokine IL-6 and a decrease in the anti-inflammatory cytokine IL-10.

### GO and KEGG Analyses Predicted Age-Related Signaling

**FIGURE 5:**
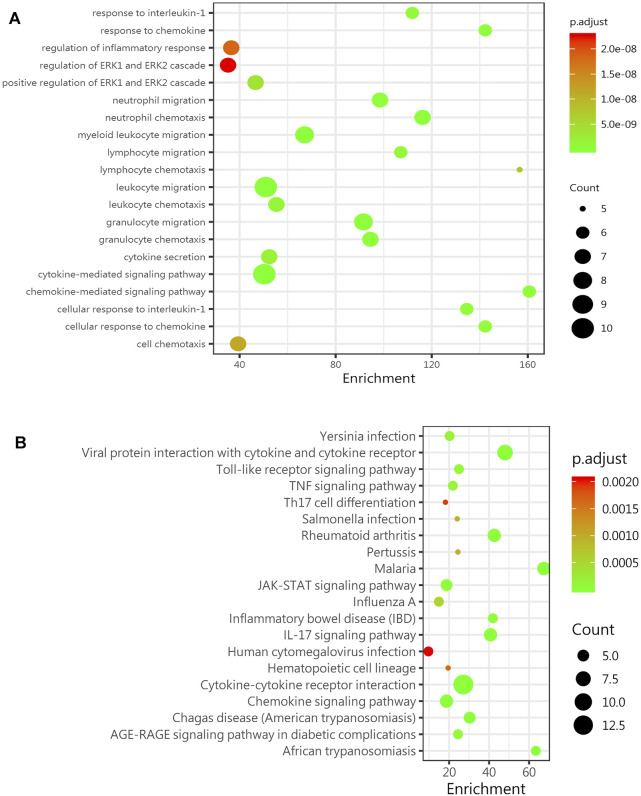
GO and KEGG analyses predict age-related signaling pathways. **(A)** Representative GO_BP (biological process) analysis of inflammatory factors in the sera of mice in the young group (8-week-old mice) and the old group (78-week-old mice) (*n* = 4). **(B)** KEGG enrichment analysis of DEGs among inflammatory factors in the sera of mice in the young group (8-week-old mice) and the old group (78-week-old mice) (*n* = 4). Abbreviations: DEGs, differentially expressed genes.

The age-related inflammatory microenvironment is characterized by an imbalance in inflammatory and anti-inflammatory pathways and is the basic mechanism of aging (Fougère et al., 2017; Franceschi et al., 2007). The GO and KEGG analyses (Figures 5A,B) were used to predict the potential mechanism. We hypothesized that these differentially expressed signaling pathways were the potential mechanism by which the BMSC functional changes were caused by the inflammatory microenvironment.

## Discussion

Chronic inflammation in the elderly population (inflammaging) is closely associated with many aging-related diseases, and an increasing number of studies have shown that stem cell therapy is a promising treatment for senile degenerative diseases (Kiernan et al., 2017; Samsonraj et al., 2017; Ding et al., 2019). Our study indicated that with increasing age, the bone mass decreased, and the number of aging cells increased. *In vitro* experiments proved that the aging microenvironment reduced the proliferation and differentiation of the BMSCs, resulting in enhanced aging characteristics in stem cells and decreased cell dryness. The inflammatory microenvironment affects many cellular physiological processes, one of which is cell senescence (Bottazzi et al., 2018). The senescent cells cannot function normally and secrete inflammatory cytokines and degradation proteins, which are called senescence-related secretory phenotype (SASP) factors (Wu et al., 2020). The SASP induces further aging and inflammation, leading to stem cell dysfunction, an aging phenotype, and chronic diseases (Kirkland and Tchkonia, 2017). The SASP is increasingly recognized as a promising therapeutic target for the prevention of age-related degenerative diseases, including osteoporosis (Farr et al., 2016). The inflammation-related factors with the most significant differences were the pro-inflammatory factor IL-6 and the anti-inflammatory factor IL-10, as screened by the protein chip technology. The changes in inflammation-related factors in the microenvironment of aging cells *in vitro* were consistent with the trend *in vivo*. The GO and KEGG analyses can lay the foundation for further mechanistic research and provide a theoretical basis and treatment strategy for stem cell therapy of age-related osteoporosis.

Many studies have shown that this age-related stem cell senescence is largely due to metabolic changes, abnormal mitochondrial activity, and impaired protein homeostasis (García-Prat et al., 2017). However, the importance of the aging pro-inflammatory environment for stem cell senescence is often neglected. A study suggested that age-related inflammation triggers skeletal stem/progenitor cell dysfunction (Josephson et al., 2019). Therefore, exploring this age-related pro-inflammatory state is of great clinical importance for the prevention and treatment of age-related osteoporosis. Our research explored the effect of the inflammatory microenvironment on the bone marrow mesenchymal stem cells *in vitro* and *in vivo*. *In vitro*, the bone marrow mesenchymal stem cells with aging characteristics were obtained through the long-term passage, and these cells were characterized by morphological changes and enhanced β-galactosidase activity (Biran et al., 2017). We found that the proliferation of cells cultured in CM-P10 was weaker than that of the cells cultured in CM-P3. From this, we observed that this age-related external environment can reduce cell proliferation and accelerate cell aging. Furthermore, we induced osteogenesis and adipogenesis in the bone marrow mesenchymal stem cells and found that the ability of the P10CM-induced stem cells to undergo osteogenesis and adipogenesis was weaker than that of P3CM-induced stem cells. It is well known that the lineage of the bone marrow cells changes with age and is characterized by an obvious reduction in osteogenesis and an increase in adipogenesis (Li et al., 2017; Infante and Rodríguez, 2018). However, other studies pointed out that the ability of the mesenchymal stem cells to undergo osteogenesis and adipogenesis was generally decreased (Bonab et al., 2006). BMSCs have different aging mechanisms *in vitro* and *in vivo*. *In vivo*, the differentiation of the bone marrow mesenchymal stem cells is strictly controlled by the local environment, which maintains a balance between osteogenesis and adipogenesis. Aging itself may provide a favorable adipogenic environment, resulting in fat accumulation in the aged bone due to the cellular resistance to oxidative stress and apoptosis. (Kim et al., 2012; Chen et al., 2016; Li et al., 2016; Ganguly et al., 2017). According to Chen et al. (2016), the *in vitro* culture environment lacks complex molecular fate control mechanisms, including transforming growth factor-β (TGF-β)/bone morphogenetic protein (BMP), Wnt and Hedgehog signals, and specific transcription factors, such as runt-related transcription factor 2 (Runx2) and peroxisome proliferator-activated receptor γ (PPARγ); thus, the balance between osteogenic and adipogenic differentiation is not maintained. According to our experimental results, we hypothesized that the aging-related inflammatory environment reduces the stemness of BMSCs, leading to the loss of differentiation ability. By examining stemness-related factors such as Nanog and OCT4, it was confirmed that the age-related inflammatory environment reduced the stemness of BMSCs.

To explore this aging inflammatory state in detail, we selected mice in different age groups and tested their sera with Quantibody^®^Mouse Infection Array 1. We found that 14 inflammatory factors and chemokines were significantly changed in the aged group compared with the young group, and most of these factors were upregulated. The two inflammation-related factors with the greatest changes were the pro-inflammatory factor IL-6 and the anti-inflammatory factors IL-10, IL-6, and IL-10 in the bone marrow, which showed the same trend. A study showed that an imbalance in IL-6 cytokine signaling contributes to the onset and maintenance of several diseases, including rheumatoid arthritis and osteoporosis. IL-6-type cytokines can lead to the activation of JAK/STAT (Janus kinase/signal transduction and transcription activator) and MAPK (mitogen activation) (Heinrich et al., 2003). Laren Becker et al. (Sui et al., 2016) showed that an increase in inflammatory factors such as IL-6, IL-1β, and IL-18 was observed in the enteric nervous system with age. Interleukin-10 (IL-10) is an anti-inflammatory cytokine with an important immunoregulatory role (Farr et al., 2017). IL-10 can block the NF-κB activity and participate in the regulation of the JAK-STAT signaling pathway. The increase in chemokines such as BLC (CXCL13), MCP-5 (CCL2), and eotaxin (CCL11) also indicates a disorder of the extracellular microenvironment. Through the GO and KEGG analyses, the JAK-STAT signaling pathway was shown to be significantly changed, laying the foundation for further research on relevant mechanisms.

Treatment strategies for osteoporosis include inhibiting stem cell senescence or eliminating the senescent cells (Jeon et al., 2017; Khosla et al., 2018). With increasing age, the number of senescent cells in the bone increases. These senescent cells produce pro-inflammatory factors, resulting in increased bone resorption and decreased bone formation (Lasry and Ben-Neriah, 2015; Sui et al., 2016). Targeting aging cells represents a novel therapeutic strategy that can prevent not only the bone loss but also a variety of age-related diseases (Lasry and Ben-Neriah, 2015; Sui et al., 2016). Studies have shown that small molecular compounds such as quercetin, rapamycin, resveratrol, and melatonin can inhibit stem cell senescence (Shuai et al., 2016; Yi et al., 2016; Li et al., 2018). Regeneration can be restored by regulating the inflammatory environment with pharmacological compounds (Hardeland, 2019). For example, JAK 1/2 inhibitors are used to inhibit the production of the pro-inflammatory SASP in aging cells (Xu et al., 2015a; Xu et al., 2015b; Wu et al., 2020). Another treatment strategy for osteoporosis is stem cell transplantation. Stem cells have a wide range of applications in basic research, and they have great potential in regenerative medicine. Stem cell therapy has been extensively studied in degenerative diseases and is a feasible treatment option (Drosos and Kolios, 2013; Holan et al., 2017). However, the limitation of stem cell transplantation is that a large number of young stem cells are needed. Understanding the inflammatory microenvironment is helpful in maintaining stem cell stemness. Some studies have shown the protective effect of melatonin on bone marrow mesenchymal stem cells during long-term passage *in vitro*. In addition to protecting stem cells and inhibiting aging, we can further explore whether the aging of stem cells can be reversed by changing the inflammatory environment to provide further support for stem cell therapy for age-related osteoporosis.

## Data Availability

The original contributions presented in the study are included in the article/Supplementary Material; further inquiries can be directed to the corresponding authors.
