# Supplementary material for: Inflammatory Microenvironment Accelerates Bone Marrow Mesenchymal Stem Cell Aging
Source: Front Bioeng Biotechnol. 2022 May 12;10:870324. doi: 10.3389/fbioe.2022.870324 (PMC9133389; doi:10.3389/fbioe.2022.870324)
Supplement: Supplementary file 1 [file DataSheet1.docx]

***Supplementary Figure***


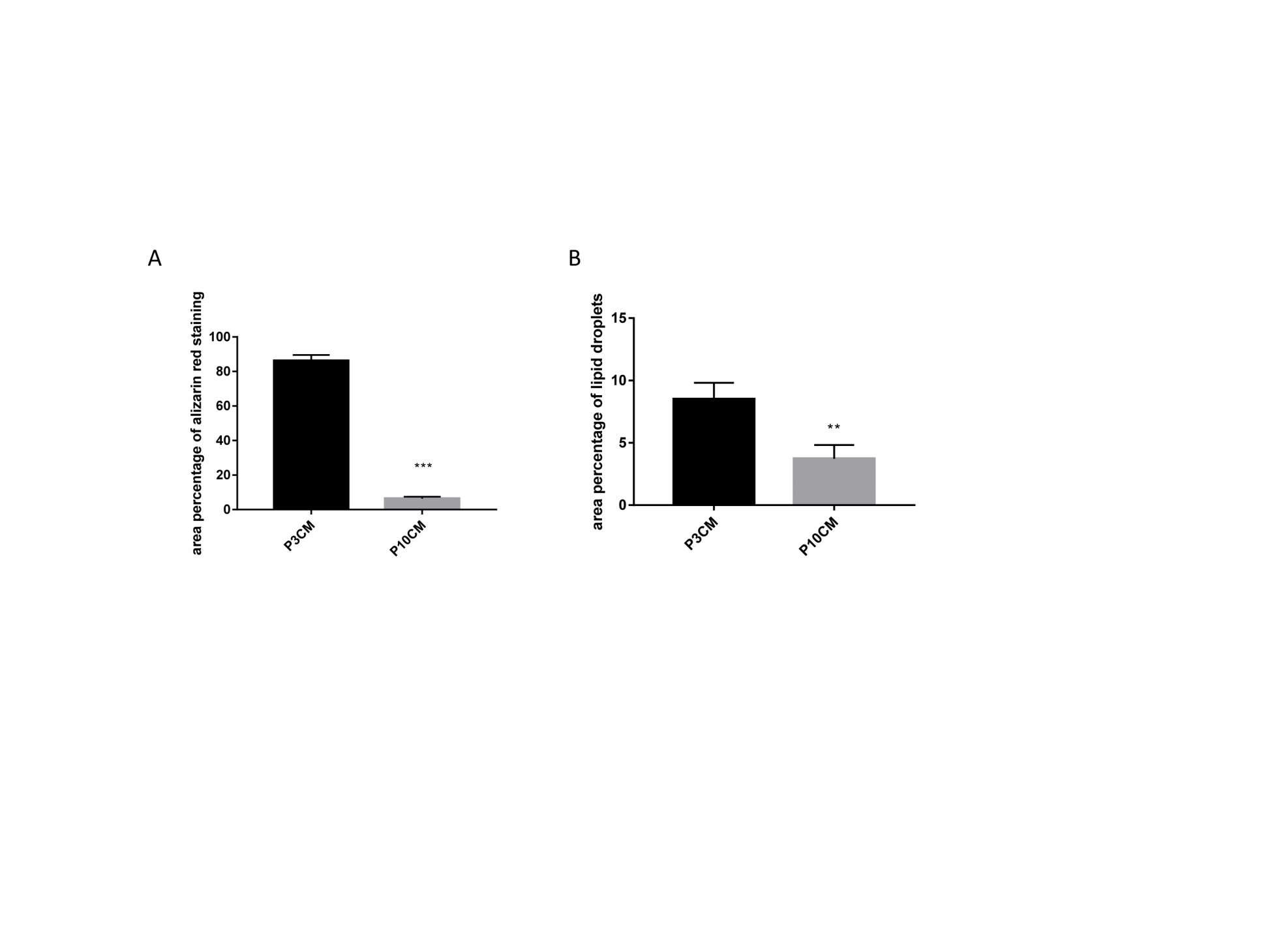


Supplementary Figure 1: Quantitative analysis of alizarin red staining and oil red O staining. Data represent the mean ± SD. **p < 0.01, and ***p < 0.001 (compared with control), from Student’s t tests.

With aging, the external microenvironment has a greater impact on the proliferation capacity of BMSCs. However, the TUNEL assay showed that the external environment in aging had no obvious effect on BMSC apoptosis (Supplementary Figure 2). This result shows that the external environment in aging mainly reduces the number of BMSCs by reducing the proliferation of BMSCs rather than inducing apoptosis.


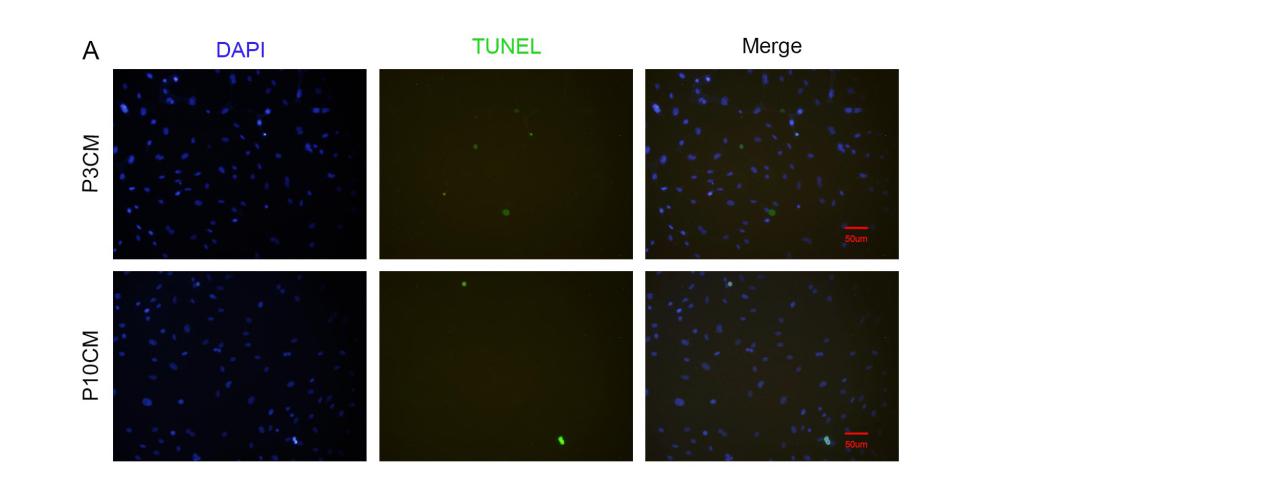


Supplementary Figure 2: The external environment in aging has no obvious effect on BMSC apoptosis. (A) TUNEL staining of BMSCs after treatment with P3 CM and P10 CM. Scale bar, 50 µm.

| proteinID | AveExp.B | AveExp.A | logFC | adj.P.V-al | Fold change | Regulation | entrezID |
| --- | --- | --- | --- | --- | --- | --- | --- |
| IL-6 | 8.09 | 4.21 | 3.88 | 0.0000 | 14.72 | up | 16193 |
| IL-10 | 0.00 | 4.78 | -4.78 | 0.0000 | 0.04 | down | 16153 |
| BLC | 9.49 | 6.85 | 2.63 | 0.0001 | 6.23 | up | 55985 |
| MCP-5 | 6.18 | 4.62 | 1.56 | 0.0015 | 2.95 | up | 20293 |
| Eoxtaxin | 9.36 | 8.91 | 0.45 | 0.0016 | 1.37 | up | 20292 |
| IL-1β | 0.09 | 2.19 | -2.09 | 0.0023 | 0.23 | down | 16176 |
| IL-17 | 4.60 | 3.27 | 1.33 | 0.0046 | 2.52 | up | 16171 |
| G-CSF | 10.97 | 7.69 | 3.28 | 0.0047 | 9.71 | up | 12985 |
| Leptin | 9.39 | 8.52 | 0.86 | 0.010 | 1.82 | up | 16846 |
| GM-CSF | 0.82 | 3.28 | -2.46 | 0.014 | 0.18 | down | 12983 |
| TCA-3 | 3.82 | 4.68 | -0.86 | 0.015 | 0.55 | down | 20190 |
| MIP-1a | 1.24 | 5.96 | -4.72 | 0.025 | 0.04 | down | 20302 |
| ICAM-1 | 1.17 | 5.50 | 4.34 | 0.026 | 0.05 | down | 15894 |
| MIG | 8.01 | 7.58 | 0.43 | 0.047 | 1.35 | up | 17329 |

Table1: Specific information on 14 inflammation-related factors with significantly different changes.
